# New Insight in Hyperinsulinism/Hyperammonemia Syndrome by Magnetic Resonance Imaging and Spectroscopy

**DOI:** 10.3390/brainsci12030389

**Published:** 2022-03-15

**Authors:** Karim Gariani, Antoine Klauser, Maria Isabel Vargas, François Lazeyras, Christel Tran

**Affiliations:** 1 Division of Endocrinology, Diabetes, Hypertension and Therapeutic Patient Education, Geneva University Hospitals, University of Geneva, 1205 Geneva, Switzerland; karim.gariani@hcuge.ch; 2 Department of Radiology and Medical Informatics, University of Geneva, 1211 Geneva, Switzerland; antoine.klauser@unige.ch (A.K.); francois.lazeyras@hcuge.ch (F.L.); 3 Center for Biomedical Imaging (CIBM), 1015 Lausanne, Switzerland; 4 Division of Neuroradiology, Geneva University Hospitals, University of Geneva, 1205 Geneva, Switzerland; maria.i.vargas@hcuge.ch; 5 Center for Molecular Diseases, Division of Genetic Medicine, University Hospital of Lausanne, University of Lausanne, 1011 Lausanne, Switzerland

**Keywords:** glutamate dehydrogenase, brain spectroscopy, hyperinsulinism, hyperammonemia

## Abstract

Hyperinsulinism/hyperammonemia syndrome (HI/HA) is an autosomal dominant disorder caused by monoallelic activating mutations in the glutamate dehydrogenase 1 (*GLUD1*) gene. While hyperinsulinism may be explained by a reduction in the allosteric inhibition of *GLUD1*, the pathogenesis of HA in HI/HA remains uncertain; interestingly, HA in the HI/HA syndrome is not associated with acute hyperammonemic intoxication events. We obtained a brain magnetic resonance (MR) in a woman with HI/HA syndrome with chronic asymptomatic HA. On MR spectroscopy, choline and myoinositol were decreased as in other HA disorders. In contrast, distinct from other HA disorders, combined glutamate and glutamine levels were normal (not increased). This observation suggests that brain biochemistry in HI/HA may differ from that of other HA disorders. In HI/HA, ammonia overproduction may come to the expense of glutamate levels, and this seems to prevent the condensation of ammonia with glutamate to produce glutamine that is typical of the other HA disorders. The absence of combined glutamate and glutamine elevation might be correlated to the absence of acute cerebral ammonia toxicity.

## 1. Introduction

The hyperinsulinism-hyperammonemia syndrome, described by Cochrane et al., in 1956 [1], is caused by monoallelic activating variants in the *GLUD1* gene (HI/HA syndrome, OMIM# 606762) that encodes the mitochondrial enzyme glutamate dehydrogenase (GDH) expressed in the liver, kidney, brain and pancreatic β-cells. It catalyzes the oxidative deamination of glutamate to α-ketoglutarate with release of free ammonia. Impaired sensitivity of GDH to GTP inhibition leads to excessive insulin secretion by pancreatic β-cells and impaired ammonia metabolism in the liver and kidney [2]. GDH-catalyzed glutamate metabolism plays important roles in 3 tissues: (1) in pancreatic β-cells, the regulation of leucine amino acid-stimulated insulin secretion; (2) in hepatocytes, the oxidative deamination of glutamate to α-ketoglutarate and ammonia; (3) in brain neurons, maintenance of glutamate neurotransmitter concentrations. HI/HA patients have protein induced hypoglycemia together with persistent, mild HA that is usually asymptomatic [3]. Although patients with HI/HA do not show the classical signs of hyperammonemia (i.e., irritability, headache, vomiting, ataxia, encephalopathy, or coma) secondary brain damage is not excluded, in particular in the developing brain. Indeed, cognitive impairment, developmental delay and various forms of seizures are reported in patients with HI-HA. The mechanism for HA in HI-HA and its impact on the brain remains unclear [4,5,6]. Seizures and coma have been explained by hypoglycemia, but both might be related to chronic HA. If this is true, the prevention of hypoglycemia may not prevent disabilities and seizures. HA may also result from other genetic conditions related to inherited metabolic disorders such as urea cycle disorders (UCDs) or organic acidurias, where a specific enzymatic defect leads to either defective ammonium removal or overproduction of ammonium [7,8]. More recently, an increase in plasma ammonium was observed in a knock-in rat model for glutaric aciduria type 1 under high lysine diet [9,10].

Magnetic resonance imaging (MRI) and spectroscopy (MRS) are used to capture information on possible mechanism of neural injury and brain metabolites [11,12]. The application of MRS in patients with chronic hepatic encephalopathy and UCD has shown abnormalities in cerebral glutamate-glutamine (Glx), myoinositol and choline metabolites, suspected to contribute to neurotoxicity [13,14].

While taking care of a woman with HI/HA, we were intrigued by the fact that she had no neurologic signs in spite of chronic HA. A workup including metabolic profiling, MRI, and magnetic resonance spectroscopic imaging (MRSI) of the brain, as well as neuropsychological evaluation, was performed.

## 2. Case Report

A 27-year-old female with HI/HA syndrome was referred to the Adult Metabolic Clinic for follow-up. She had a history of hypoglycemia since early infancy that was treated with diazoxide. Chronic asymptomatic HA (between 145 and 204 μmol/L, normal range 15–35) was observed that did not respond to standard therapies (i.e., nitrogen scavenger and antibiotics [15]). Her history was relevant for learning disabilities, but she had no history of coma, seizure or altered level of consciousness. Gene sequencing of *GLUD1* had previously shown a monoallelic pathogenic variant (p.Gly499Val) (Division of Genetic Medicine, Geneva University Hospitals [16]).

Neurological examination was essentially normal with no phasic trouble or dysarthria. Cranial nerves and cerebellar examination were also normal and no defects in sensory and motor functions were observed. Psychometric examination using Montreal cognitive Assessment (MoCA) test version 8.1 revealed mild executive and attention defects without impact on daily activity. Plasma ammonia was elevated at 190 µmol/L (normal range 15–35 µmol/L). Liver enzyme tests were normal. Plasma amino acids were measured using high-performance liquid chromatography (Service of Clinical Chemistry, University Hospital of Lausanne) and showed borderline low glutamine (398 µmol/L, normal range 440–810), glutamate (36 µmol/L, normal range 20–70), arginine (63 µmol/L, normal range 25–125), and citrulline (13 µmol/L, normal range 55–55). Urine organic acids showed slight elevation of lactate, Krebs cycle intermediates and glutamate. Orotic acid was undetectable. Plasma free carnitine was borderline high (22 µmol/L, normal range 5.6–20.9).

**Figure 1:**
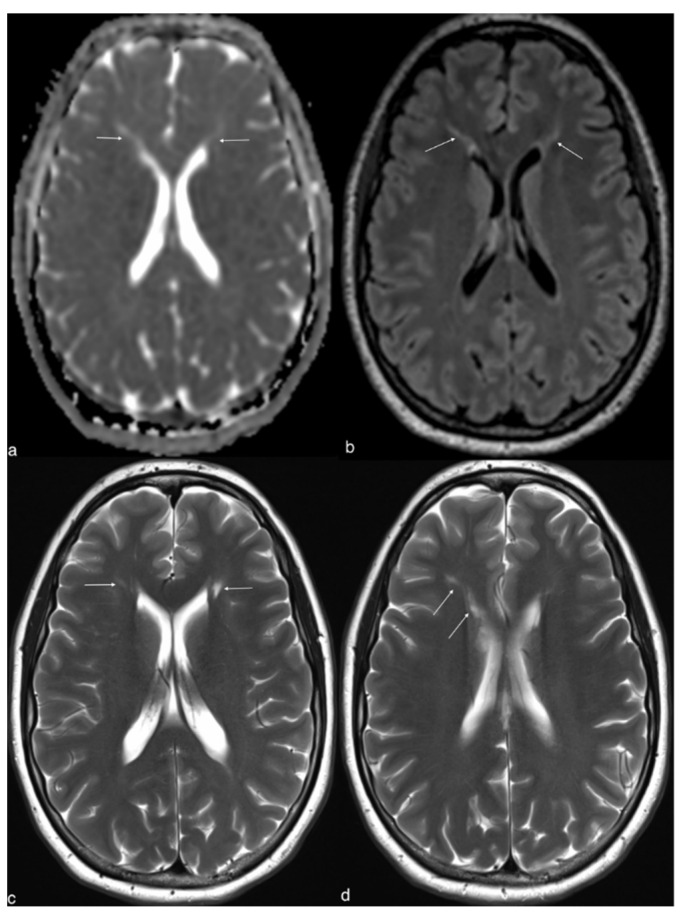
Brain MRI shows an increased ADC (arrows in (**a**)) associated with linear high signals lesions of the white matter in front of the frontal horns of lateral ventricles on FLAIR and T2 (arrows in (**b**–**d**)). ADC, apparent diffusion coefficient; MRI, magnetic resonance imaging.

A brain MRI bilaterally showed areas of increased apparent diffusion coefficient (ADC) value, hyperintensity on T2-weighted and fluid-attenuated inversion-recovery (FLAIR), of the white matter, radially oriented and extended from the frontal ventricular horns toward the periphery with no connection with the cortex (Figure 1).

**Figure 2:**
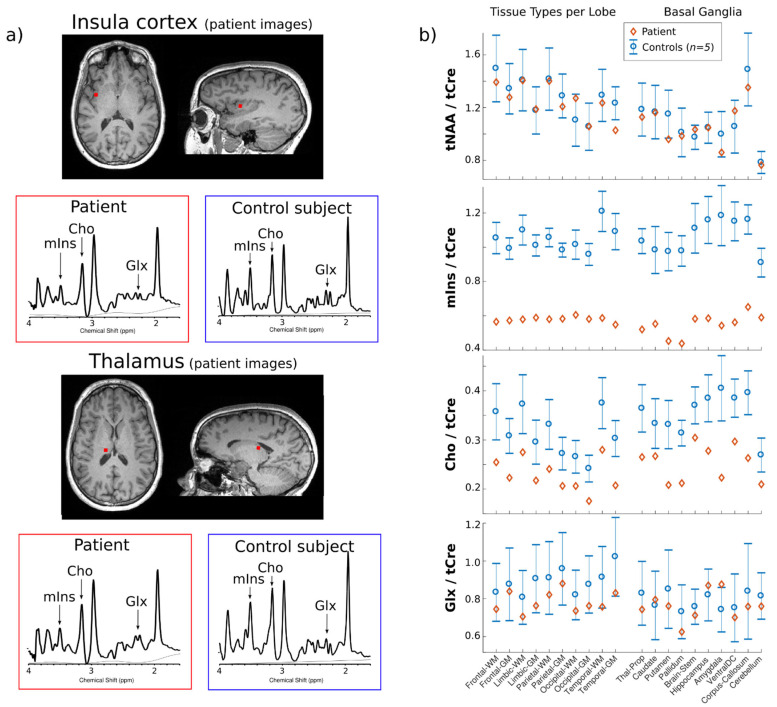
MRSI spectra measured in the insula cortex and the thalamus of the patient (corresponding voxel in red shown on anatomical T1-weighted images) (**a**). For comparison, spectra from the same structures are shown for a healthy subject. The drop of choline containing compounds (Cho) and inositol (Ins) in peak intensity is particularly visible in the patient spectra whereas the glutamate + glutamine (Glx) peaks show no clear alteration. These observations are confirmed by an analysis of the metabolite concentration over the whole patient brain (**b**). Regional quantitative values are shown for the patient (red diamonds) and the controls (blue circles represent the mean of the 5 with the standard deviation as error bar). The metabolite concentration ratios Ins/creatine + phosphocreatine (tCre) and Cho/tCre show systematic and distinct lower levels in the patient compared to the control group. N-acetylaspartate + N-acetyl aspartylglutamate (tNAA)/tCre and glutamate + glutamine (Glx)/tCre show no clear difference. MRSI, magnetic resonance spectroscopic imaging.

MRSI with 5-mm isotropic resolution [17] (TR 355 ms, TE 0.65 ms, quantification with LCModel, http://s-provencher.com/lcmodel.shtml, accessed on 11 January 2022) was performed over the patient whole brain and compared to 5 controls (Figure 2a). *N*-acetylaspartate + *N*-acetyl aspartylglutamate (tNAA), creatine + phosphocreatine (tCre), choline moiety metabolites (Cho) (choline, acetylcholine, phosphocholine and glycerophosphocholine), myoinositol (mIns), and glutamate + glutamine (Glx) were measured against data from 5 controls in the anatomical structures of the brain (Figure 2b). This showed a markedly reduced mIns/tCre ratio (−45% in average overall structures, Z-score = −5.5) and Cho/tCre ratio (−26%, Z-score = −2.1) over all brain structures. Surprisingly other metabolites (tNAA/tCre ratio and Glx/tCre ratio) showed no difference with control values (Z-score = −0.2, −0.16, respectively).

## 3. Discussion

HI/HA was initially described as a disorder of glucose homeostasis, but patients with HI/HA also have chronic HA, seizures, and neurodevelopmental disabilities that currently are not well-understood and for which no specific treatment has been proposed. To obtain an insight into these unanswered questions, we performed brain imaging as well as MRSI in a patient with HI/HA and HA. Interestingly, we observed some new features.

The brain MRI showed signal abnormalities and involvement of white matter that are reminiscent of those reported in the literature in patients with HA. Although we cannot ignore that this might be attributed to other factors such as hypoxic injury or recurrent hypoglycemia, it is plausible that the pattern in our patient may be directly related to HA [18,19,20]. Ammonia toxicity in the brain is tightly connected to glutamine metabolism [21,22,23]. This observation is supported by MRS that has shown high glutamine concentrations during HA in rat models [24,25] but also in human with UCD or hepatic encephalopathy (Table 1) [13,14,26,27,28,29,30,31,32,33,34,35,36,37].

In contrast, MRS in our patient showed normal level of Glx compared to controls together with borderline low plasma glutamine level. 

**Figure 3:**
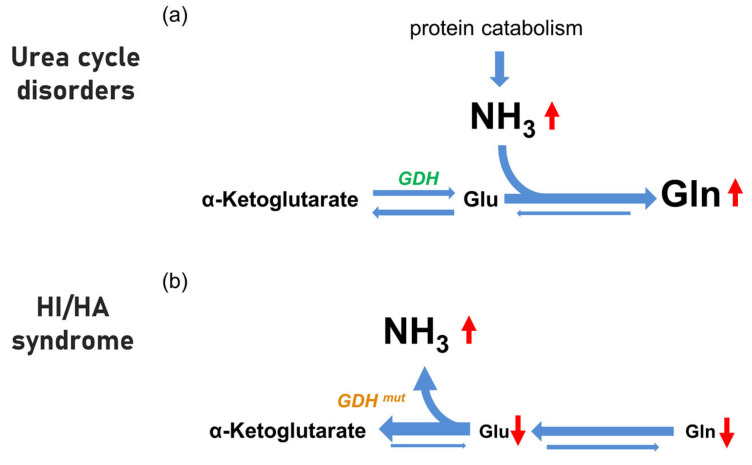
Schematic of the major pathways by which cerebral glutamate (Glu) and glutamine (Gln) levels are affected in urea cycle disorders (**a**) and hypothesis for HI/HA syndrome (**b**) related to increased activity of glutamate dehydrogenase (GDH). Relative changes in pool size of cerebral metabolites (α-ketoglutarate, ammonia, glutamate, and glutamine) between different conditions are indicated by differences in font size. In (**a**) ammonia (NH_3_) levels are primarily increased, and they drive the condensation reaction with glutamate to produce glutamine by glutamine synthetase; the α-ketoglutarate -glutamate-glutamine pathway is shifted towards to the right. In (**b**) we hypothesize that in HI/HA, the activating mutations in the GDH enzyme drive the deamination of glutamate to produce ammonia and α-ketoglutarate; thus, the αketoglutarate-glutamate-glutamine pathway is driven towards the left, and there is no accumulation of glutamine. (**a**) adapted from [42]). HA, hyperammonemia; HI, hyperinsulinism.

It is currently believed that in HI/HA, overactivity of GDH in the brain and liver leads to sustained release of ammonia from glutamate oxidation and secondarily to depletion of the pools of glutamine and glutamate [2,38,39,40,41]. In contrast, in UCD, the primary defect in ammonia detoxification leads to an increase (rather than a decrease) in the glutamine pool and, as a consequence, to osmotic shifts between the intracellular and extracellular compartments [41,42,43]. In other words, in UCD, glutamate is available and is used by glutamine synthetase to detoxify the ammonia; the net production of glutamine is highly increased. In HI/HA, we hypothesize that the equilibrium is shifted in the opposite direction: increased GDH activity leads to glutamate depletion with formation of ammonia and alpha-ketoglutarate, and because of glutamate depletion, glutamine is not increased. Thus, even at comparable levels of ammonia, the difference between UCDs and the HI/HA syndrome is that in the former, glutamine is elevated, and in the latter, glutamine is normal. The absence of elevated glutamine (and of the secondary osmotic shifts) might explain the absence of acute brain toxicity in the HI/HA syndrome (Figure 3). 

Nonetheless, other indirect consequences of chronic HA are not excluded [4]. In addition, MRSI showed decreased choline and myoinositol compared to controls (Figure 2). Myoinositol functions as a marker of astrocyte activity while choline relates to membrane turnover [14,36]. Both metabolites have been shown to decrease in HA disorders such as UCD or hepatic encephalopathy and might be surrogate markers of neuronal suffering [37]. Whether this is related to HA and may contribute to brain injury in HI/HA remains hypothetical but deserves attention in terms of biochemical changes in the brain. The whole-brain MRSI technique used in this study measures the metabolic content of the patient’s entire brain in a single scan [17], distinct from the traditional MRSI approach that is limited to a specific volume of interest [11]. Thus, it is possible to monitor the alteration of metabolites in all brain structures at once as demonstrated on the Figure 2. This should be beneficial for the diagnosis and monitoring of patients in clinical practice.

The combined measurement of Glx would fail to detect slight and opposite alterations in glutamate and glutamine levels. Nevertheless, a significant increase in glutamine as measured in patients with UCD or hepatic encephalopathy would result in significant increase in Glx. In the future, MR spectroscopy at ultra-high field (>7 Tesla) might allow one to distinguish glutamine resonance from glutamate due to the greater chemical shift dispersion and would provide a deeper understanding of the neurochemical environment in the HI/HA disease.

## 4. Conclusions

The level of Glx measured with MRSI over the whole brain of this HI/HA patient is different from those known through UCD patients. In particular, the lack of Glx elevation might imply the absence of cytotoxic osmotic changes and thus explain the absence of acute cerebral ammonia toxicity in HI/HA. Higher field MRSI studies allowing for the separation between Glu and Gln will be needed to confirm or refute this hypothesis.

## Figures and Tables

**Table 1:** Examples of brain MRI and MRS findings in adult patients with hyperammonemia due to urea cycle disorders, cirrhosis-related hepatic encephalopathy and drug-induced encephalopathy.

Disease	MRI Findings	MRS Findings	Ref.
OTC	NA	Elevated glutamine in posterior cingulate gray matter, parietal and frontal WM. Reduction in myoinositol and choline in parietal and frontal white matter, thalamus and posterior cingulate gray matter	[36]
OTC	Increased signal on T2-weighted and diffusion-weighted images in the basal ganglia, claustrum, frontoparietal WM, pontine tegmentum, and left brachium pontis	Elevated glutamine. Reduction in myoinositol and choline	[29]
OTC	No structural abnormalities in gray or white matter	Elevated glutamine and glutamate. Reduction in myoinositol and choline	[37]
Type II citrullinemia	Bilateral, non-enhancing abnormalities of the globus pallidus, insular cortex, and cingulate gyrus on T2-weighted and diffusion-weighted MRI	Elevated glutamine and glutamate. Reduction in myoinositol and choline	[26]
Hepatic encephalopathy	Elevated apparent diffusion coefficient values in the corticospinal tract and parietal white matter	Elevated glutamine, reduced myoinositol and choline and non-significant difference in glutamate and *N*-acetylaspartate	[33]
Hepatic encephalopathy	NA	Elevated glutamine and glutamate. Reduction in myoinositol and choline	[27]
Hepatic encephalopathy	Occipital white matter and basal ganglia had significant hyperintensity	Elevated glutamine and glutamate. Reduction in myoinositol and choline	[34]
Valproate-induced encephalopathy	Metabolic-toxic lesion pattern with bilateral T2-hyperintense lesion in the cerebellar white matter and in the globus pallidus	Elevated glutamine and glutamate. Reduction in myoinositol and choline	[35]

Abbreviations: MRI, magnetic resonance imaging; MRS, magnetic resonance spectroscopy; NA, not available; OTC, Ornithine transcarbamylase deficiency; WM, white matter.

## Data Availability

The data presented in this study are available on request from the corresponding author. The data are not publicly available due to privacy reason.
